# Crystal structure of levomepromazine maleate

**DOI:** 10.1107/S2056989016004916

**Published:** 2016-04-05

**Authors:** Gyula Tamás Gál, Nóra Veronika May, Petra Bombicz

**Affiliations:** a Institute of Organic Chemistry, Research Centre for Natural Sciences, Hungarian Academy of Sciences, H-1519 Budapest, POB 206, Hungary

**Keywords:** crystal structure, pheno­thia­zine, maleate, hydrogen bonding, C—H⋯π inter­actions

## Abstract

The asymmetric unit of the title salt, C_19_H_25_N_2_OS^+^·C_4_H_3_O_4_^−^ [systematic name: (*S*)-3-(2-meth­oxy­pheno­thia­zin-10-yl)-*N*,*N*,2-tri­methyl­propanaminium hydrogen maleate], comprises two (*S*)-levomepromazine cations and two hydrogen maleate anions. The conformations of the two cations are similar. The major difference relates to the orientation of the meth­oxy substituent at the pheno­thia­zine ring system. The crystal components form a three-dimensional supra­molecular network *via* N—H⋯O, C—H⋯O and C—H⋯π inter­actions. A comparison of the conformations of the levomepromazine cations with those of the neutral mol­ecule and similar protonated mol­ecules reveals significant conformational flexibility of the pheno­thia­zine ring system and the substituent at the pheno­thia­zine N atom.

## Abstract 2

In the crystal, enanti­omerically pure (*S*)-3-(2-meth­oxy­pheno­thia­zin-10-yl)-*N*,*N*,2-tri­methyl­propanaminium hydrogen maleate, also known as levomepromazine maleate, forms a three-dimensional supra­molecular network through N—H⋯O, C—H⋯O and C—H⋯π inter­actions. The asymmetric unit comprises two slightly conformationally different levomepromazine cations and two hydrogen maleate anions.

## Chemical context   

Levomepromazine maleate is a type of tranquilizer that is widely used as an important active pharmaceutical ingredient (API). As a typical N-substituted pheno­thia­zine anti­psychotic, this API is able to block a variety of receptors. For example, levomepromazine is used for treating schizophrenia (Froim­owitz & Cody, 1993 ▸). The levomepromazine mol­ecule is chiral and the (*R*)-(−) enanti­omer is the medically active form. It is worth noting that the neutral (*R)-*levomepromazine mol­ecule corresponds to the (*S*)-levomepromazine cation formed by protonation of its tertiary amino group, according to the Cahn–Ingold-Prelog (CIP) convention. The crystal structure of neutral (*R)-*levomepromazine has been reported previously, including the determination of its absolute configuration (Sato e*t al.*). As (*R)-*levomepromazine is generally sold in the form of its maleate salt, we report here the crystal structure of this compound and compare the conformation of neutral levomepromazine with those of its cationic forms.
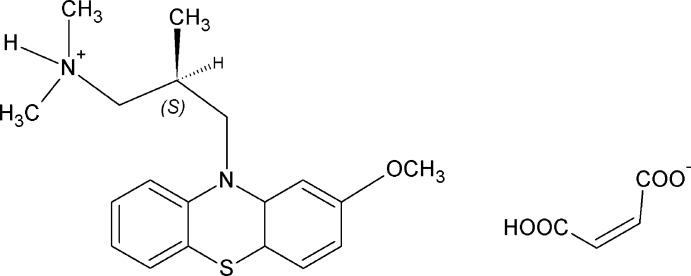


## Structural commentary   

**Figure 1:**
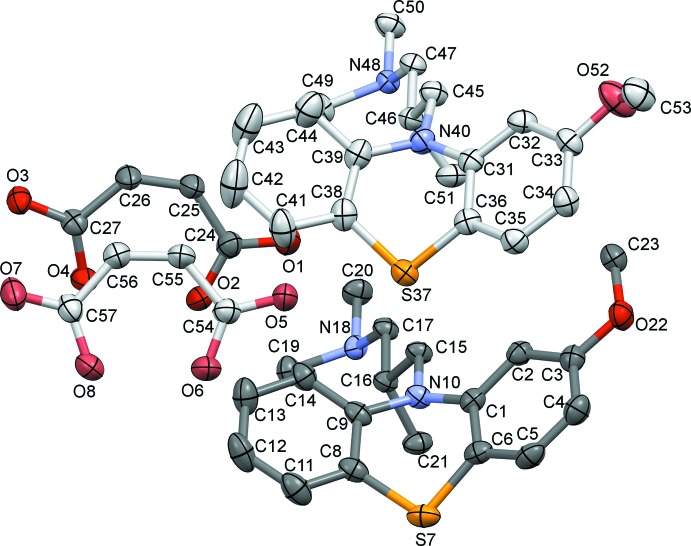
The mol­ecular structure of the title compound, showing the atom labelling. Displacement ellipsoids are drawn at the 50% probability level. The asymmetric unit contains two organic salt mol­ecules. H atoms have been omitted for clarity.

**Figure 2:**
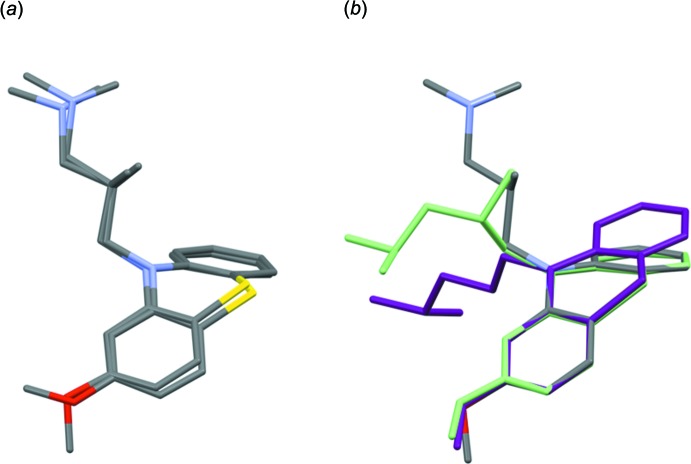
Conformational comparison of (*a*) the two levomepromazine mol­ecules in the asymmetric unit of the title structure, and (*b*) one of the levomepromazines from the title structure (gray) compared with neutral dimorphic levomepromazine (green, MPZPAM) as well as the non-methyl­ated derivative (purple, MAPTML10).

The asymmetric unit of the title compound comprises two levomepromazine cations and two hydrogen maleate anions (Fig. 1 ▸). The nitro­gen atoms N18 and N48 are protonated, thus the cations contain a tertiary amine group. The main difference in the cationic structures results from the different orientation of the meth­oxy substituent of the pheno­thia­zine ring system, as illustrated in Fig. 2 ▸ *a* where superposition of the two cations is presented. The root-mean-square deviation measuring the average distance between the atoms of the superimposed mol­ecules is 0.509 Å and the maximum distance between the meth­oxy carbon atoms is 2.980 (4) Å. The pheno­thia­zine groups are similarly bent along the N—S line with dihedral angles between the benzene rings of 42.51 (17) and 43.71 (18)°; these values are close to the analogous dihedral angles in the neutral levomepromazine mol­ecule [41.24° at room temperature (MPZPAM; Sato *et al.*, 1980 ▸) and 43.09° at 121 K (Dahl *et al.*, 1982 ▸)].

The conformation of the investigated levomepromazine hydrogen maleate salt was compared with that of neutral levomepromazin (MPZPAM) and with the closely related compound 3-(2-meth­oxy-10-pheno­thia­zin­yl)-*N*,*N*-dimethyl-propanaminium hydrogen maleate, in which the propyl side chain is non-methyl­ated (MAPTML10; Marsau & Gauthier, 1973 ▸) (see Fig. 2 ▸ *b*). Mol­ecules MPZPAM and MAPTML10 were inverted to obtain the same conformation for the pheno­thia­zine rings (which resulted in the opposite enanti­o­mer for MPZPAM). It can be seen that the main difference is in the torsion angle around the N10—C15 bond and the conformation of the side chain. For MPZPAM, the pheno­thia­zine ring could be fully superimposed with the pheno­thia­zine ring of the title compound, but the propyl side chains differ in the configuration and orientation of their amino­methyl groups. In the non-methyl­ated derivative MAPTML10, the heterocyclic ring system is significantly closer to being flat (the dihedral angle between the benzene rings is 21.74°), while the aliphatic chain bends to the opposite site of the pheno­thia­zine ring in comparison with the title compound.

**Figure 3:**
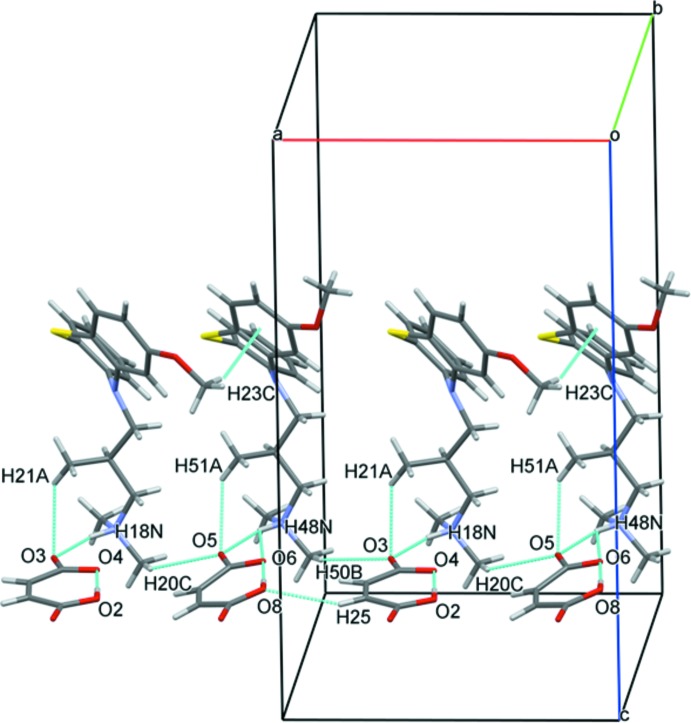
The view of the columnar structure arrangement extending along the *a* axis showing the C—H⋯O and C—H⋯π inter­actions as turquoise lines.

The planar structure of the hydrogen maleate anions is stabilized by very strong intra­molecular O—H⋯O hydrogen bonds between the carb­oxy­lic and carboxyl­ate groups, as is often observed for these anions (Table 1 ▸, Fig. 3 ▸).

## Supra­molecular features   

**Figure 4:**
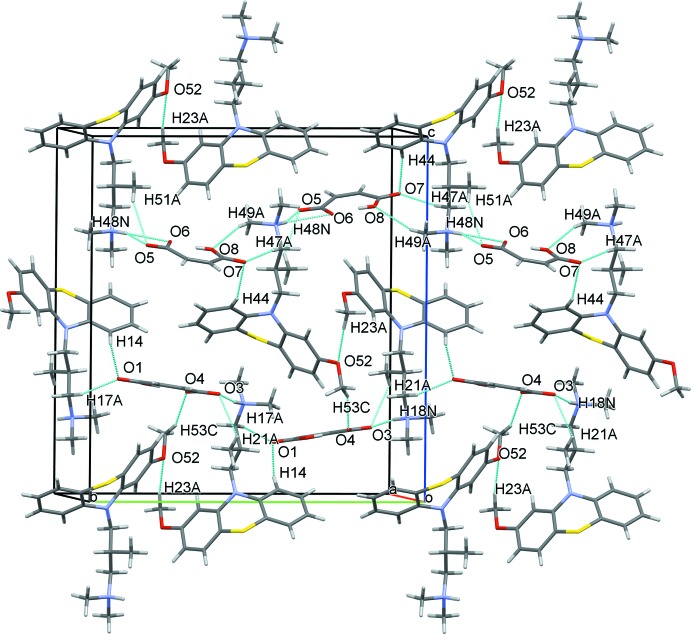
Crystal packing along the *bc* plane showing the N—H⋯O and C—H⋯O inter­actions as turquoise lines.

The crystal structure of the title compound features strong N—H⋯O hydrogen bonds and several weak C—H⋯O inter­actions (Table 1 ▸). The maleate anions form ionic pairs with the protonated amino groups of the levomepromazine cations by strong N—H⋯O inter­actions (Fig. 3 ▸). The meth­oxy groups of the levomepromazine cations differ in their inter­molecular inter­actions. In one, the meth­oxy methyl group is involved in a C—H⋯π inter­action to the aromatic ring of a neighbouring levomepromazine cation [C23—H23*C*⋯*Cg*(C31–C36), Table 1 ▸]. The same methyl group forms an additional hydrogen bond to a meth­oxy O atom of the other symmetry-independent levomepromazine cation (C23—H23*A*⋯O52, Fig. 4 ▸). There are numerous C—H⋯O inter­actions between the hydrogen maleate anions and the levomepromazine C—H groups, assisting the assembly of the crystal components in the *bc* plane (Table 1 ▸, Fig. 4 ▸).

## Synthesis and crystallization   

The title compound was obtained from EGIS Pharmaceuticals Private Limited Company and used without further purification. The compound was enanti­omerically pure, its melting point is 457–459 K. Colorless single crystals were obtained by slow evaporation of the solvent from an ethyl acetate solution over one week.

## Refinement   

Crystal data, data collection and details of the structure refinement are summarized in Table 2 ▸. The 13 missing reflections were found to be obstructed by the beamstop. All H atoms were located in difference electron-density maps. Hydrogen atoms were included in the structure-factor calculations but they were not refined; their positions were calculated with C—H = 0.95–1.00 Å and they were allowed to ride on their parent atoms, with *U*_iso_(H) = 1.2*U*_eq_(C) for aromatic, methyl­ene and methine and *U*_iso_(H) = 1.5*U*_eq_(C) for methyl protons. The absolute configuration around the C16 and C46 atoms in the title compound (Fig. 1 ▸) were determined to be *S* from anomalous dispersion effects.

## Supplementary Material

Crystal structure: contains datablock(s) I. DOI: 10.1107/S2056989016004916/gk2652sup1.cif

Structure factors: contains datablock(s) I. DOI: 10.1107/S2056989016004916/gk2652Isup2.hkl

Supporting information file. DOI: 10.1107/S2056989016004916/gk2652Isup3.cml

CCDC reference: 1470232

Additional supporting information:  crystallographic information; 3D view; checkCIF report

## Figures and Tables

**Table 1:** Hydrogen-bond geometry (Å, °) *Cg* is the centroid of the C31–C36 ring.

*D*—H⋯*A*	*D*—H	H⋯*A*	*D*⋯*A*	*D*—H⋯*A*
O2—H2*O*⋯O4	0.84	1.61	2.452 (3)	178
O8—H8*O*⋯O6	0.84	1.61	2.443 (4)	174
N18—H18*N*⋯O3^i^	1.00	1.73	2.716 (3)	170
N48—H48*N*⋯O5^ii^	1.00	1.74	2.710 (3)	164
N48—H48*N*⋯O6^ii^	1.00	2.63	3.332 (4)	128
C11—H11⋯O3^iii^	0.95	2.52	3.316 (5)	141
C14—H14⋯O1	0.95	2.53	3.466 (5)	167
C17—H17*A*⋯O1	0.99	2.43	3.340 (4)	153
C19—H19*B*⋯O7^iii^	0.99	2.49	3.449 (5)	166
C23—H23*A*⋯O52^iv^	0.98	2.56	3.518 (5)	167
C23—H23*C*⋯*Cg*	0.98	2.47	3.421 (4)	145
C47—H47*A*⋯O7^v^	0.99	2.34	3.296 (4)	161
C49—H49*B*⋯O1	0.98	2.39	3.333 (5)	163
C50—H50*B*⋯O3^vi^	0.98	2.55	3.278 (4)	131
C53—H53*C*⋯O4^vii^	0.98	2.56	3.479 (5)	156

Symmetry codes: (i) 
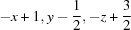
; (ii) 
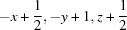
; (iii) 
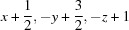
; (iv) 
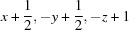
; (v) 
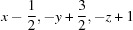
; (vi) 
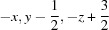
; (vii) 
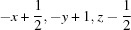
.

**Table 2:** Experimental details

Crystal data	
Chemical formula	C_19_H_25_N_2_OS^+^·C_4_H_3_O_4_^−^
*M*_r_	444.53
Crystal system, space group	Orthorhombic, *P*2_1_2_1_2_1_
Temperature (K)	103
*a*, *b*, *c* (Å)	11.6395 (5), 19.0487 (6), 20.4977 (7)
*V* (Å^3^)	4544.7 (3)
*Z*	8
Radiation type	Mo *K*α
μ (mm^−1^)	0.18
Crystal size (mm)	0.5 × 0.3 × 0.2

Data collection	
Diffractometer	R-AXIS RAPID
Absorption correction	Numerical *NUMABS*; Higashi, 2002 ▸
*T*_min_, *T*_max_	0.893, 0.971
No. of measured, independent and observed [*I* > 2σ(*I*)] reflections	105320, 10363, 8459
*R*_int_	0.085
(sin θ/λ)_max_ (Å^−1^)	0.649

Refinement	
*R*[*F* ^2^ > 2σ(*F* ^2^)], *wR*(*F* ^2^), *S*	0.049, 0.133, 1.05
No. of reflections	10363
No. of parameters	569
H-atom treatment	H-atom parameters constrained
Δρ_max_, Δρ_min_ (e Å^−3^)	0.67, −0.31
Absolute structure	Flack *x* determined using 3169 quotients [(*I* ^+^)−(*I* ^−^)]/[(*I* ^+^)+(*I* ^−^)] (Parsons *et al.*, 2013 ▸
Absolute structure parameter	−0.02 (3)

Computer programs: *CrystalClear* (Rigaku/MSC, 2007 ▸), *SHELXS97* (Sheldrick, 2008 ▸), *SHELXL2014* (Sheldrick, 2015 ▸), *Mercury* (Macrae *et al.*, 2006 ▸) and *PLATON* (Spek, 2009 ▸).

## supplementary crystallographic information

### Crystal data

**Table:**

C_19_H_25_N_2_OS^+^·C_4_H_3_O_4_^−^	*D*_x_ = 1.299 Mg m^−^^3^
*M**_r_* = 444.53	Melting point = 457–459 K
Orthorhombic, *P*2_1_2_1_2_1_	Mo *K*α radiation, λ = 0.71073 Å
*a* = 11.6395 (5) Å	Cell parameters from 74983 reflections
*b* = 19.0487 (6) Å	θ = 3.2–27.5°
*c* = 20.4977 (7) Å	µ = 0.18 mm^−^^1^
*V* = 4544.7 (3) Å^3^	*T* = 103 K
*Z* = 8	Prism, colourless
*F*(000) = 1888	0.5 × 0.3 × 0.2 mm

### Data collection

**Table:**

R-AXIS RAPID diffractometer	10363 independent reflections
Radiation source: NORMAL-focus sealed tube	8459 reflections with *I* > 2σ(*I*)
Graphite monochromator	*R*_int_ = 0.085
Detector resolution: 10.0000 pixels mm^-1^	θ_max_ = 27.5°, θ_min_ = 3.2°
dtprofit.ref scans	*h* = −15→15
Absorption correction: numerical *NUMABS*; Higashi, 2002	*k* = −24→24
*T*_min_ = 0.893, *T*_max_ = 0.971	*l* = −26→26
105320 measured reflections	

### Refinement

**Table:**

Refinement on *F*^2^	Secondary atom site location: structure-invariant direct methods
Least-squares matrix: full	Hydrogen site location: difference Fourier map
*R*[*F*^2^ > 2σ(*F*^2^)] = 0.049	H-atom parameters constrained
*wR*(*F*^2^) = 0.133	*w* = 1/[σ^2^(*F*_o_^2^) + (0.0791*P*)^2^] where *P* = (*F*_o_^2^ + 2*F*_c_^2^)/3
*S* = 1.05	(Δ/σ)_max_ < 0.001
10363 reflections	Δρ_max_ = 0.67 e Å^−^^3^
569 parameters	Δρ_min_ = −0.31 e Å^−^^3^
0 restraints	Absolute structure: Flack *x* determined using 3169 quotients [(*I*^+^)-(*I*^-^)]/[(*I*^+^)+(*I*^-^)] (Parsons *et al.*, 2013
Primary atom site location: structure-invariant direct methods	Absolute structure parameter: −0.02 (3)

### Special details

**Table:**

Geometry. All esds (except the esd in the dihedral angle between two l.s. planes) are estimated using the full covariance matrix. The cell esds are taken into account individually in the estimation of esds in distances, angles and torsion angles; correlations between esds in cell parameters are only used when they are defined by crystal symmetry. An approximate (isotropic) treatment of cell esds is used for estimating esds involving l.s. planes.

### Fractional atomic coordinates and isotropic or equivalent isotropic displacement parameters (Å^2^)

**Table:**

	*x*	*y*	*z*	*U*_iso_*/*U*_eq_	
S7	0.71907 (8)	0.43498 (5)	0.42513 (4)	0.0376 (2)	
S37	0.27962 (8)	0.47770 (4)	0.44305 (4)	0.0324 (2)	
O3	0.2810 (2)	0.85941 (11)	0.69965 (12)	0.0340 (5)	
O4	0.4194 (2)	0.77876 (12)	0.69694 (13)	0.0380 (6)	
O5	0.2773 (2)	0.64259 (12)	0.29296 (13)	0.0395 (6)	
O1	0.3156 (3)	0.56393 (12)	0.65973 (14)	0.0458 (7)	
O2	0.4351 (2)	0.65270 (12)	0.67442 (14)	0.0397 (6)	
H2O	0.4307	0.6961	0.6812	0.048*	
O22	0.3315 (2)	0.23474 (12)	0.42581 (12)	0.0367 (6)	
O7	0.3143 (3)	0.93613 (13)	0.33972 (16)	0.0521 (8)	
O6	0.4133 (2)	0.72465 (13)	0.28657 (14)	0.0428 (6)	
N48	0.0515 (2)	0.44623 (13)	0.75804 (13)	0.0267 (6)	
H48N	0.1129	0.4098	0.7628	0.032*	
N18	0.5491 (2)	0.45179 (13)	0.77062 (12)	0.0261 (6)	
H18N	0.6141	0.4180	0.7763	0.031*	
O8	0.4289 (2)	0.85049 (13)	0.30770 (15)	0.0441 (6)	
H8O	0.4253	0.8067	0.3030	0.053*	
N10	0.5392 (2)	0.43086 (14)	0.52619 (13)	0.0274 (6)	
C16	0.5607 (3)	0.44213 (16)	0.64667 (14)	0.0256 (6)	
H16	0.5808	0.4929	0.6417	0.031*	
C20	0.4693 (3)	0.4429 (2)	0.82668 (16)	0.0351 (8)	
H20A	0.4062	0.4767	0.8230	0.053*	
H20B	0.5110	0.4509	0.8675	0.053*	
H20C	0.4380	0.3951	0.8264	0.053*	
C27	0.3141 (3)	0.79688 (17)	0.69359 (16)	0.0304 (7)	
C17	0.4879 (3)	0.43375 (17)	0.70818 (14)	0.0269 (6)	
H17A	0.4192	0.4641	0.7042	0.032*	
H17B	0.4611	0.3845	0.7108	0.032*	
N40	0.0636 (3)	0.45554 (14)	0.51572 (13)	0.0327 (6)	
C36	0.1707 (3)	0.41769 (16)	0.42030 (15)	0.0288 (7)	
C46	0.0698 (3)	0.44388 (16)	0.63495 (15)	0.0291 (7)	
H46	0.1011	0.4928	0.6358	0.035*	
C45	−0.0030 (3)	0.43515 (18)	0.57258 (16)	0.0331 (7)	
H45A	−0.0276	0.3856	0.5682	0.040*	
H45B	−0.0727	0.4647	0.5756	0.040*	
C47	−0.0078 (3)	0.43418 (17)	0.69381 (16)	0.0290 (7)	
H47A	−0.0733	0.4671	0.6902	0.035*	
H47B	−0.0391	0.3859	0.6932	0.035*	
C50	−0.0309 (3)	0.4373 (2)	0.81310 (17)	0.0358 (8)	
H50A	0.0107	0.4396	0.8546	0.054*	
H50B	−0.0691	0.3917	0.8093	0.054*	
H50C	−0.0885	0.4748	0.8116	0.054*	
C14	0.5155 (3)	0.55931 (18)	0.53450 (17)	0.0339 (7)	
H14	0.4561	0.5537	0.5659	0.041*	
C21	0.6714 (3)	0.39977 (18)	0.64729 (17)	0.0327 (7)	
H21A	0.7201	0.4156	0.6834	0.049*	
H21B	0.7121	0.4065	0.6059	0.049*	
H21C	0.6532	0.3499	0.6529	0.049*	
O52	−0.0838 (3)	0.27161 (18)	0.38223 (15)	0.0592 (9)	
C15	0.4825 (3)	0.42064 (17)	0.58918 (14)	0.0270 (7)	
H15A	0.4609	0.3706	0.5939	0.032*	
H15B	0.4112	0.4489	0.5905	0.032*	
C1	0.5213 (3)	0.38102 (15)	0.47535 (15)	0.0264 (6)	
C9	0.5686 (3)	0.50069 (17)	0.50747 (15)	0.0299 (7)	
C24	0.3317 (3)	0.62659 (17)	0.66822 (18)	0.0341 (8)	
C54	0.3118 (3)	0.70422 (17)	0.30093 (18)	0.0347 (8)	
C33	0.0037 (3)	0.31867 (17)	0.39121 (17)	0.0332 (7)	
C19	0.5978 (3)	0.52445 (17)	0.76919 (17)	0.0339 (8)	
H19A	0.5370	0.5580	0.7580	0.051*	
H19B	0.6589	0.5269	0.7364	0.051*	
H19C	0.6293	0.5361	0.8122	0.051*	
C3	0.4162 (3)	0.28458 (16)	0.42653 (17)	0.0319 (7)	
C26	0.2222 (3)	0.74344 (17)	0.68253 (19)	0.0366 (8)	
H26	0.1462	0.7617	0.6829	0.044*	
C6	0.5962 (3)	0.38091 (17)	0.42268 (17)	0.0313 (7)	
C55	0.2258 (4)	0.75469 (18)	0.32921 (19)	0.0401 (8)	
H55	0.1546	0.7342	0.3416	0.048*	
C57	0.3310 (3)	0.87372 (18)	0.32830 (18)	0.0358 (8)	
C39	0.0910 (3)	0.52782 (18)	0.50652 (17)	0.0362 (8)	
C31	0.0745 (3)	0.41109 (16)	0.46133 (15)	0.0283 (7)	
C8	0.6538 (3)	0.51034 (18)	0.46007 (16)	0.0325 (7)	
C4	0.4893 (4)	0.28620 (18)	0.37300 (18)	0.0378 (8)	
H4	0.4784	0.2541	0.3380	0.045*	
C34	0.0976 (3)	0.32530 (18)	0.35056 (17)	0.0345 (8)	
H34	0.1053	0.2961	0.3132	0.041*	
C5	0.5772 (3)	0.33408 (18)	0.37057 (17)	0.0360 (8)	
H5	0.6259	0.3357	0.3334	0.043*	
C38	0.1890 (3)	0.54566 (17)	0.47043 (16)	0.0348 (8)	
C13	0.5500 (4)	0.62664 (18)	0.51517 (19)	0.0444 (10)	
H13	0.5144	0.6667	0.5340	0.053*	
C2	0.4303 (3)	0.33317 (16)	0.47697 (16)	0.0294 (7)	
H2	0.3779	0.3337	0.5125	0.035*	
C25	0.2291 (3)	0.67395 (17)	0.67218 (18)	0.0360 (8)	
H25	0.1572	0.6511	0.6664	0.043*	
C51	0.1706 (3)	0.3927 (2)	0.63410 (19)	0.0381 (8)	
H51A	0.2198	0.4014	0.6720	0.057*	
H51B	0.2151	0.3994	0.5940	0.057*	
H51C	0.1415	0.3445	0.6357	0.057*	
C56	0.2338 (3)	0.82429 (18)	0.33969 (19)	0.0391 (8)	
H56	0.1666	0.8453	0.3572	0.047*	
C32	−0.0088 (3)	0.36124 (17)	0.44602 (17)	0.0327 (7)	
H32	−0.0746	0.3563	0.4731	0.039*	
C23	0.2465 (3)	0.23796 (17)	0.47604 (18)	0.0347 (8)	
H23A	0.2832	0.2313	0.5186	0.052*	
H23B	0.1894	0.2009	0.4690	0.052*	
H23C	0.2085	0.2839	0.4749	0.052*	
C49	0.1073 (3)	0.51674 (18)	0.76233 (18)	0.0363 (8)	
H49A	0.0502	0.5533	0.7534	0.054*	
H49B	0.1694	0.5197	0.7302	0.054*	
H49C	0.1388	0.5234	0.8062	0.054*	
C35	0.1809 (3)	0.37539 (17)	0.36501 (16)	0.0323 (7)	
H35	0.2453	0.3808	0.3370	0.039*	
C12	0.6355 (4)	0.6354 (2)	0.46897 (19)	0.0477 (10)	
H12	0.6589	0.6812	0.4564	0.057*	
C11	0.6869 (4)	0.5769 (2)	0.44106 (18)	0.0433 (9)	
H11	0.7448	0.5827	0.4089	0.052*	
C42	0.1473 (6)	0.6686 (2)	0.4844 (2)	0.0651 (15)	
H42	0.1664	0.7163	0.4767	0.078*	
C43	0.0522 (5)	0.6517 (2)	0.5214 (2)	0.0640 (14)	
H43	0.0068	0.6880	0.5399	0.077*	
C44	0.0220 (4)	0.5815 (2)	0.5320 (2)	0.0522 (11)	
H44	−0.0450	0.5703	0.5564	0.063*	
C41	0.2149 (5)	0.61537 (18)	0.45858 (19)	0.0482 (10)	
H41	0.2796	0.6270	0.4325	0.058*	
C53	−0.1196 (4)	0.2632 (2)	0.3167 (2)	0.0514 (10)	
H53A	−0.1352	0.3093	0.2975	0.077*	
H53B	−0.1896	0.2346	0.3154	0.077*	
H53C	−0.0588	0.2397	0.2918	0.077*	

### Atomic displacement parameters (Å^2^)

**Table:**

	*U*^11^	*U*^22^	*U*^33^	*U*^12^	*U*^13^	*U*^23^
S7	0.0263 (4)	0.0496 (5)	0.0369 (4)	0.0003 (4)	0.0064 (4)	0.0061 (4)
S37	0.0307 (4)	0.0340 (4)	0.0325 (4)	−0.0046 (3)	−0.0002 (4)	0.0012 (3)
O3	0.0333 (13)	0.0272 (11)	0.0415 (13)	0.0013 (10)	−0.0098 (11)	0.0008 (9)
O4	0.0258 (13)	0.0347 (12)	0.0535 (16)	−0.0009 (10)	−0.0049 (11)	0.0017 (11)
O5	0.0373 (14)	0.0298 (12)	0.0513 (15)	−0.0017 (11)	0.0075 (13)	−0.0054 (10)
O1	0.0445 (17)	0.0293 (13)	0.0636 (17)	0.0004 (11)	0.0159 (13)	−0.0005 (12)
O2	0.0287 (14)	0.0324 (12)	0.0581 (17)	0.0044 (10)	0.0049 (12)	−0.0010 (11)
O22	0.0408 (15)	0.0330 (11)	0.0362 (13)	−0.0048 (11)	−0.0023 (11)	−0.0072 (10)
O7	0.0460 (18)	0.0310 (13)	0.079 (2)	−0.0045 (12)	−0.0112 (15)	−0.0009 (13)
O6	0.0294 (14)	0.0404 (13)	0.0586 (17)	−0.0002 (11)	0.0101 (13)	−0.0012 (12)
N48	0.0252 (14)	0.0248 (12)	0.0302 (13)	−0.0022 (11)	0.0021 (11)	0.0001 (10)
N18	0.0259 (14)	0.0291 (13)	0.0234 (12)	0.0003 (11)	−0.0018 (11)	−0.0038 (10)
O8	0.0327 (15)	0.0389 (13)	0.0608 (18)	−0.0084 (11)	0.0039 (13)	0.0008 (13)
N10	0.0279 (15)	0.0328 (13)	0.0214 (12)	−0.0046 (11)	0.0011 (11)	−0.0005 (11)
C16	0.0224 (16)	0.0298 (15)	0.0246 (14)	−0.0028 (13)	−0.0001 (12)	−0.0016 (12)
C20	0.037 (2)	0.0455 (19)	0.0231 (16)	−0.0010 (16)	0.0044 (13)	−0.0042 (14)
C27	0.0304 (19)	0.0321 (16)	0.0288 (16)	−0.0012 (13)	−0.0072 (13)	0.0050 (13)
C17	0.0237 (16)	0.0325 (15)	0.0245 (15)	−0.0044 (13)	−0.0020 (12)	−0.0029 (12)
N40	0.0352 (17)	0.0339 (14)	0.0289 (14)	−0.0013 (12)	0.0042 (12)	−0.0038 (11)
C36	0.0308 (17)	0.0317 (15)	0.0238 (15)	0.0024 (13)	−0.0029 (13)	0.0042 (12)
C46	0.0240 (17)	0.0308 (15)	0.0324 (16)	−0.0022 (13)	0.0021 (13)	−0.0014 (13)
C45	0.0303 (18)	0.0381 (16)	0.0309 (17)	−0.0026 (14)	0.0018 (14)	−0.0013 (14)
C47	0.0219 (15)	0.0326 (15)	0.0325 (16)	−0.0027 (13)	−0.0013 (13)	0.0000 (13)
C50	0.0325 (19)	0.0436 (19)	0.0312 (17)	−0.0068 (15)	0.0048 (14)	0.0007 (15)
C14	0.037 (2)	0.0341 (16)	0.0305 (17)	−0.0053 (15)	−0.0017 (14)	0.0002 (14)
C21	0.0250 (17)	0.0432 (18)	0.0297 (17)	0.0027 (14)	−0.0025 (14)	−0.0052 (14)
O52	0.060 (2)	0.0741 (19)	0.0432 (16)	−0.0270 (17)	−0.0057 (15)	−0.0035 (14)
C15	0.0255 (17)	0.0344 (15)	0.0210 (15)	−0.0042 (13)	0.0027 (12)	−0.0017 (12)
C1	0.0275 (17)	0.0288 (15)	0.0228 (15)	0.0044 (12)	−0.0012 (12)	−0.0008 (12)
C9	0.0267 (17)	0.0370 (17)	0.0259 (15)	−0.0075 (14)	−0.0056 (13)	0.0028 (13)
C24	0.036 (2)	0.0293 (16)	0.0374 (19)	0.0021 (14)	0.0060 (15)	0.0043 (14)
C54	0.031 (2)	0.0356 (18)	0.0371 (19)	0.0005 (14)	0.0047 (15)	0.0014 (14)
C33	0.0325 (19)	0.0354 (17)	0.0316 (17)	−0.0067 (15)	−0.0037 (15)	0.0030 (14)
C19	0.039 (2)	0.0303 (16)	0.0319 (17)	−0.0043 (15)	−0.0036 (14)	−0.0055 (14)
C3	0.0346 (19)	0.0288 (15)	0.0321 (17)	0.0013 (13)	−0.0033 (15)	−0.0018 (13)
C26	0.0250 (17)	0.0302 (16)	0.055 (2)	0.0010 (14)	−0.0056 (17)	0.0003 (14)
C6	0.0275 (17)	0.0351 (16)	0.0313 (17)	0.0073 (13)	0.0022 (14)	0.0022 (13)
C55	0.030 (2)	0.0370 (18)	0.053 (2)	−0.0032 (15)	0.0113 (18)	−0.0049 (15)
C57	0.035 (2)	0.0315 (17)	0.041 (2)	−0.0039 (15)	−0.0073 (16)	0.0028 (14)
C39	0.045 (2)	0.0327 (17)	0.0311 (17)	0.0061 (15)	−0.0009 (15)	−0.0005 (14)
C31	0.0281 (18)	0.0297 (15)	0.0270 (16)	0.0018 (13)	−0.0033 (13)	0.0018 (12)
C8	0.0283 (18)	0.0422 (18)	0.0269 (16)	−0.0073 (14)	−0.0021 (13)	0.0046 (13)
C4	0.046 (2)	0.0362 (18)	0.0314 (18)	0.0051 (16)	0.0009 (16)	−0.0069 (14)
C34	0.043 (2)	0.0328 (16)	0.0275 (16)	−0.0044 (15)	−0.0001 (15)	−0.0009 (13)
C5	0.038 (2)	0.0404 (18)	0.0295 (17)	0.0089 (15)	0.0084 (15)	−0.0010 (14)
C38	0.047 (2)	0.0311 (16)	0.0264 (16)	−0.0013 (15)	−0.0032 (15)	0.0021 (13)
C13	0.065 (3)	0.0323 (17)	0.036 (2)	−0.0061 (18)	−0.0023 (19)	−0.0037 (15)
C2	0.0294 (18)	0.0313 (15)	0.0276 (16)	0.0034 (13)	−0.0006 (14)	−0.0029 (13)
C25	0.0261 (18)	0.0308 (16)	0.051 (2)	−0.0015 (14)	−0.0002 (17)	0.0022 (14)
C51	0.0250 (18)	0.048 (2)	0.041 (2)	0.0015 (15)	0.0041 (16)	−0.0017 (16)
C56	0.033 (2)	0.0351 (17)	0.050 (2)	0.0008 (15)	0.0056 (17)	−0.0034 (15)
C32	0.0249 (17)	0.0408 (17)	0.0324 (17)	−0.0003 (14)	−0.0015 (14)	0.0001 (14)
C23	0.036 (2)	0.0326 (16)	0.0355 (18)	−0.0058 (14)	−0.0015 (15)	−0.0003 (14)
C49	0.039 (2)	0.0307 (17)	0.0396 (19)	−0.0105 (15)	−0.0014 (15)	−0.0005 (14)
C35	0.0341 (19)	0.0342 (16)	0.0286 (17)	−0.0003 (14)	0.0033 (14)	0.0031 (13)
C12	0.068 (3)	0.040 (2)	0.035 (2)	−0.0235 (19)	−0.004 (2)	0.0051 (16)
C11	0.050 (2)	0.049 (2)	0.0305 (18)	−0.0188 (17)	−0.0003 (16)	0.0023 (16)
C42	0.117 (5)	0.0329 (19)	0.045 (2)	0.002 (2)	0.008 (3)	−0.0007 (18)
C43	0.100 (4)	0.039 (2)	0.053 (3)	0.019 (2)	0.015 (3)	−0.0016 (19)
C44	0.064 (3)	0.047 (2)	0.046 (2)	0.009 (2)	0.008 (2)	−0.0054 (18)
C41	0.074 (3)	0.0338 (18)	0.037 (2)	−0.0100 (19)	0.005 (2)	0.0015 (15)
C53	0.052 (3)	0.060 (2)	0.043 (2)	−0.013 (2)	−0.0028 (19)	−0.0103 (19)

### Geometric parameters (Å, º)

**Table:**

S7—C6	1.763 (4)	O52—C53	1.416 (5)
S7—C8	1.775 (4)	C15—H15A	0.9900
S37—C38	1.761 (4)	C15—H15B	0.9900
S37—C36	1.769 (4)	C1—C6	1.388 (5)
O3—C27	1.258 (4)	C1—C2	1.398 (5)
O4—C27	1.276 (4)	C9—C8	1.401 (5)
O5—C54	1.252 (4)	C24—C25	1.499 (5)
O1—C24	1.221 (4)	C54—C55	1.504 (5)
O2—C24	1.309 (5)	C33—C34	1.380 (5)
O2—H2O	0.8400	C33—C32	1.393 (5)
O22—C3	1.369 (4)	C19—H19A	0.9800
O22—C23	1.429 (4)	C19—H19B	0.9800
O7—C57	1.227 (4)	C19—H19C	0.9800
O6—C54	1.277 (4)	C3—C4	1.389 (5)
N48—C50	1.491 (4)	C3—C2	1.397 (5)
N48—C49	1.494 (4)	C26—C25	1.343 (5)
N48—C47	1.504 (4)	C26—H26	0.9500
N48—H48N	1.0000	C6—C5	1.409 (5)
N18—C20	1.487 (4)	C55—C56	1.346 (5)
N18—C19	1.496 (4)	C55—H55	0.9500
N18—C17	1.504 (4)	C57—C56	1.490 (5)
N18—H18N	1.0000	C39—C38	1.401 (5)
O8—C57	1.293 (5)	C39—C44	1.402 (5)
O8—H8O	0.8400	C31—C32	1.393 (5)
N10—C1	1.425 (4)	C8—C11	1.382 (5)
N10—C9	1.426 (4)	C4—C5	1.372 (5)
N10—C15	1.463 (4)	C4—H4	0.9500
C16—C21	1.520 (5)	C34—C35	1.392 (5)
C16—C17	1.527 (4)	C34—H34	0.9500
C16—C15	1.544 (4)	C5—H5	0.9500
C16—H16	1.0000	C38—C41	1.383 (5)
C20—H20A	0.9800	C13—C12	1.383 (6)
C20—H20B	0.9800	C13—H13	0.9500
C20—H20C	0.9800	C2—H2	0.9500
C27—C26	1.494 (5)	C25—H25	0.9500
C17—H17A	0.9900	C51—H51A	0.9800
C17—H17B	0.9900	C51—H51B	0.9800
N40—C31	1.406 (4)	C51—H51C	0.9800
N40—C39	1.426 (4)	C56—H56	0.9500
N40—C45	1.453 (4)	C32—H32	0.9500
C36—C35	1.396 (5)	C23—H23A	0.9800
C36—C31	1.406 (5)	C23—H23B	0.9800
C46—C47	1.519 (4)	C23—H23C	0.9800
C46—C51	1.525 (5)	C49—H49A	0.9800
C46—C45	1.543 (5)	C49—H49B	0.9800
C46—H46	1.0000	C49—H49C	0.9800
C45—H45A	0.9900	C35—H35	0.9500
C45—H45B	0.9900	C12—C11	1.387 (6)
C47—H47A	0.9900	C12—H12	0.9500
C47—H47B	0.9900	C11—H11	0.9500
C50—H50A	0.9800	C42—C43	1.379 (8)
C50—H50B	0.9800	C42—C41	1.388 (7)
C50—H50C	0.9800	C42—H42	0.9500
C14—C9	1.391 (5)	C43—C44	1.399 (6)
C14—C13	1.401 (5)	C43—H43	0.9500
C14—H14	0.9500	C44—H44	0.9500
C21—H21A	0.9800	C41—H41	0.9500
C21—H21B	0.9800	C53—H53A	0.9800
C21—H21C	0.9800	C53—H53B	0.9800
O52—C33	1.369 (4)	C53—H53C	0.9800
			
C6—S7—C8	97.88 (16)	N18—C19—H19A	109.5
C38—S37—C36	97.47 (17)	N18—C19—H19B	109.5
C24—O2—H2O	109.5	H19A—C19—H19B	109.5
C3—O22—C23	117.5 (2)	N18—C19—H19C	109.5
C50—N48—C49	109.7 (3)	H19A—C19—H19C	109.5
C50—N48—C47	110.5 (3)	H19B—C19—H19C	109.5
C49—N48—C47	112.8 (2)	O22—C3—C4	116.6 (3)
C50—N48—H48N	107.9	O22—C3—C2	123.5 (3)
C49—N48—H48N	107.9	C4—C3—C2	119.9 (3)
C47—N48—H48N	107.9	C25—C26—C27	130.8 (3)
C20—N18—C19	110.9 (3)	C25—C26—H26	114.6
C20—N18—C17	109.6 (3)	C27—C26—H26	114.6
C19—N18—C17	112.0 (2)	C1—C6—C5	119.5 (3)
C20—N18—H18N	108.1	C1—C6—S7	119.1 (3)
C19—N18—H18N	108.1	C5—C6—S7	121.2 (3)
C17—N18—H18N	108.1	C56—C55—C54	130.1 (4)
C57—O8—H8O	109.5	C56—C55—H55	114.9
C1—N10—C9	117.4 (3)	C54—C55—H55	114.9
C1—N10—C15	119.4 (3)	O7—C57—O8	122.2 (3)
C9—N10—C15	118.0 (3)	O7—C57—C56	117.5 (4)
C21—C16—C17	114.1 (3)	O8—C57—C56	120.2 (3)
C21—C16—C15	111.4 (3)	C38—C39—C44	119.1 (3)
C17—C16—C15	106.0 (2)	C38—C39—N40	119.1 (3)
C21—C16—H16	108.4	C44—C39—N40	121.7 (4)
C17—C16—H16	108.4	C32—C31—N40	121.7 (3)
C15—C16—H16	108.4	C32—C31—C36	118.7 (3)
N18—C20—H20A	109.5	N40—C31—C36	119.5 (3)
N18—C20—H20B	109.5	C11—C8—C9	120.9 (3)
H20A—C20—H20B	109.5	C11—C8—S7	120.6 (3)
N18—C20—H20C	109.5	C9—C8—S7	118.5 (3)
H20A—C20—H20C	109.5	C5—C4—C3	120.0 (3)
H20B—C20—H20C	109.5	C5—C4—H4	120.0
O3—C27—O4	123.0 (3)	C3—C4—H4	120.0
O3—C27—C26	116.2 (3)	C33—C34—C35	119.1 (3)
O4—C27—C26	120.8 (3)	C33—C34—H34	120.5
N18—C17—C16	114.6 (3)	C35—C34—H34	120.5
N18—C17—H17A	108.6	C4—C5—C6	120.7 (3)
C16—C17—H17A	108.6	C4—C5—H5	119.7
N18—C17—H17B	108.6	C6—C5—H5	119.7
C16—C17—H17B	108.6	C41—C38—C39	120.2 (4)
H17A—C17—H17B	107.6	C41—C38—S37	121.3 (3)
C31—N40—C39	117.2 (3)	C39—C38—S37	118.5 (2)
C31—N40—C45	121.6 (3)	C12—C13—C14	120.7 (4)
C39—N40—C45	118.9 (3)	C12—C13—H13	119.7
C35—C36—C31	120.1 (3)	C14—C13—H13	119.7
C35—C36—S37	121.7 (3)	C3—C2—C1	120.2 (3)
C31—C36—S37	118.1 (2)	C3—C2—H2	119.9
C47—C46—C51	112.9 (3)	C1—C2—H2	119.9
C47—C46—C45	108.6 (3)	C26—C25—C24	130.5 (3)
C51—C46—C45	110.1 (3)	C26—C25—H25	114.8
C47—C46—H46	108.4	C24—C25—H25	114.8
C51—C46—H46	108.4	C46—C51—H51A	109.5
C45—C46—H46	108.4	C46—C51—H51B	109.5
N40—C45—C46	110.0 (3)	H51A—C51—H51B	109.5
N40—C45—H45A	109.7	C46—C51—H51C	109.5
C46—C45—H45A	109.7	H51A—C51—H51C	109.5
N40—C45—H45B	109.7	H51B—C51—H51C	109.5
C46—C45—H45B	109.7	C55—C56—C57	130.5 (4)
H45A—C45—H45B	108.2	C55—C56—H56	114.7
N48—C47—C46	113.8 (3)	C57—C56—H56	114.7
N48—C47—H47A	108.8	C33—C32—C31	120.4 (3)
C46—C47—H47A	108.8	C33—C32—H32	119.8
N48—C47—H47B	108.8	C31—C32—H32	119.8
C46—C47—H47B	108.8	O22—C23—H23A	109.5
H47A—C47—H47B	107.7	O22—C23—H23B	109.5
N48—C50—H50A	109.5	H23A—C23—H23B	109.5
N48—C50—H50B	109.5	O22—C23—H23C	109.5
H50A—C50—H50B	109.5	H23A—C23—H23C	109.5
N48—C50—H50C	109.5	H23B—C23—H23C	109.5
H50A—C50—H50C	109.5	N48—C49—H49A	109.5
H50B—C50—H50C	109.5	N48—C49—H49B	109.5
C9—C14—C13	119.6 (3)	H49A—C49—H49B	109.5
C9—C14—H14	120.2	N48—C49—H49C	109.5
C13—C14—H14	120.2	H49A—C49—H49C	109.5
C16—C21—H21A	109.5	H49B—C49—H49C	109.5
C16—C21—H21B	109.5	C34—C35—C36	120.6 (3)
H21A—C21—H21B	109.5	C34—C35—H35	119.7
C16—C21—H21C	109.5	C36—C35—H35	119.7
H21A—C21—H21C	109.5	C13—C12—C11	119.7 (3)
H21B—C21—H21C	109.5	C13—C12—H12	120.1
C33—O52—C53	114.9 (3)	C11—C12—H12	120.1
N10—C15—C16	111.9 (3)	C8—C11—C12	120.0 (4)
N10—C15—H15A	109.2	C8—C11—H11	120.0
C16—C15—H15A	109.2	C12—C11—H11	120.0
N10—C15—H15B	109.2	C43—C42—C41	119.6 (4)
C16—C15—H15B	109.2	C43—C42—H42	120.2
H15A—C15—H15B	107.9	C41—C42—H42	120.2
C6—C1—C2	119.6 (3)	C42—C43—C44	120.7 (4)
C6—C1—N10	118.6 (3)	C42—C43—H43	119.7
C2—C1—N10	121.9 (3)	C44—C43—H43	119.7
C14—C9—C8	119.1 (3)	C43—C44—C39	119.6 (4)
C14—C9—N10	122.3 (3)	C43—C44—H44	120.2
C8—C9—N10	118.6 (3)	C39—C44—H44	120.2
O1—C24—O2	121.8 (3)	C38—C41—C42	120.7 (4)
O1—C24—C25	118.3 (3)	C38—C41—H41	119.7
O2—C24—C25	119.9 (3)	C42—C41—H41	119.7
O5—C54—O6	123.5 (3)	O52—C53—H53A	109.5
O5—C54—C55	115.8 (3)	O52—C53—H53B	109.5
O6—C54—C55	120.6 (3)	H53A—C53—H53B	109.5
O52—C33—C34	124.6 (3)	O52—C53—H53C	109.5
O52—C33—C32	114.3 (3)	H53A—C53—H53C	109.5
C34—C33—C32	121.1 (3)	H53B—C53—H53C	109.5

### Hydrogen-bond geometry (Å, º)

Cg is the centroid of the C31–C36 ring.

**Table:**

*D*—H···*A*	*D*—H	H···*A*	*D*···*A*	*D*—H···*A*
O2—H2*O*···O4	0.84	1.61	2.452 (3)	178
O8—H8*O*···O6	0.84	1.61	2.443 (4)	174
N18—H18*N*···O3^i^	1.00	1.73	2.716 (3)	170
N48—H48*N*···O5^ii^	1.00	1.74	2.710 (3)	164
N48—H48*N*···O6^ii^	1.00	2.63	3.332 (4)	128
C11—H11···O3^iii^	0.95	2.52	3.316 (5)	141
C14—H14···O1	0.95	2.53	3.466 (5)	167
C17—H17*A*···O1	0.99	2.43	3.340 (4)	153
C19—H19*B*···O7^iii^	0.99	2.49	3.449 (5)	166
C23—H23*A*···O52^iv^	0.98	2.56	3.518 (5)	167
C23—H23*C*···*Cg*	0.98	2.47	3.421 (4)	145
C47—H47*A*···O7^v^	0.99	2.34	3.296 (4)	161
C49—H49*B*···O1	0.98	2.39	3.333 (5)	163
C50—H50*B*···O3^vi^	0.98	2.55	3.278 (4)	131
C53—H53*C*···O4^vii^	0.98	2.56	3.479 (5)	156

Symmetry codes: (i) −*x*+1, *y*−1/2, −*z*+3/2; (ii) −*x*+1/2, −*y*+1, *z*+1/2; (iii) *x*+1/2, −*y*+3/2, −*z*+1; (iv) *x*+1/2, −*y*+1/2, −*z*+1; (v) *x*−1/2, −*y*+3/2, −*z*+1; (vi) −*x*, *y*−1/2, −*z*+3/2; (vii) −*x*+1/2, −*y*+1, *z*−1/2.

## References

[bb1] Dahl, S. G., Hjorth, M. & Hough, E. (1982). *Mol. Pharmacol.* **21**, 409–414. 6124878

[bb2] Froimowitz, M. & Cody, V. (1993). *J. Med. Chem.* **36**, 2219–2227. 10.1021/jm00067a019 8101879

[bb3] Higashi, T. (2002). *NUMABS*. Rigaku/MSC Inc., Tokyo, Japan.

[bb4] Macrae, C. F., Edgington, P. R., McCabe, P., Pidcock, E., Shields, G. P., Taylor, R., Towler, M. & van de Streek, J. (2006). *J. Appl. Cryst.* **39**, 453–457.

[bb5] Marsau, P. & Gauthier, J. (1973). *Acta Cryst.* B**29**, 992–998.

[bb6] Parsons, S., Flack, H. D. & Wagner, T. (2013). *Acta Cryst.* B**69**, 249–259. 10.1107/S2052519213010014 PMC3661305 23719469

[bb7] Rigaku/MSC (2007). *CrystalClear*. Rigaku/MSC Inc., Tokyo, Japan.

[bb8] Sato, M., Miki, K., Tanaka, N., Kasai, N., Ishimaru, T. & Munakata, T. (1980). *Acta Cryst.* B**36**, 2176–2178.

[bb9] Sheldrick, G. M. (2008). *Acta Cryst.* A**64**, 112–122. 10.1107/S0108767307043930 18156677

[bb10] Sheldrick, G. M. (2015). *Acta Cryst.* C**71**, 3–8.

[bb11] Spek, A. L. (2009). *Acta Cryst.* D**65**, 148–155. 10.1107/S090744490804362X PMC2631630 19171970

